# Examining Provincial HPV Vaccination Schemes in Canada: Should We Standardise the Grade of Vaccination or the Number of Doses?

**DOI:** 10.1155/2015/170236

**Published:** 2015-04-12

**Authors:** Carley Rogers, Robert J. Smith?

**Affiliations:** ^1^Department of Mathematics, The University of Ottawa, 585 King Edward Avenue, Ottawa, ON, Canada K1N 6N5; ^2^Department of Mathematics and Faculty of Medicine, The University of Ottawa, 585 King Edward Avenue, Ottawa, ON, Canada K1N 6N5

## Abstract

Human papillomavirus (HPV) infection is the most common sexually transmitted infection, which is linked to several cancers and genital warts. Depending on the Canadian province, the quadrivalent vaccine is given to girls in grades 4 through 10 with either a two- or three-dose schedule. We use a mathematical model to address the following research questions: (1) Does the grade at which the girls are vaccinated significantly affect the outcome of the program? (2) What coverage rate must the provinces reach in order to reduce the impact of HPV on the Canadian population? (3) What are the implications of vaccinating with two versus three doses? The model suggests the grade of vaccination and the number of doses do not make a significant difference to the outcome of the public vaccination program. The most significant factor is the coverage rate of children and adults. We recommend that provinces vaccinate as early as possible to avoid vaccine failure due to previous infection. We also recommend that the main focus of the program should be on obtaining a large enough coverage rate for children and/or adults in order to achieve the desired outcome with either two or three doses of the vaccine.

## 1. Introduction

Human papillomavirus (HPV) is the most common sexually transmitted virus, infecting 75% of the sexually active Canadian population [1, 2]. It can cause cancer of the cervix, vulva, oropharynx, penis, and anus, as well as genital warts [3–5]. Until recently, there were no medical interventions against HPV infections except monitoring the virus's progress through the Papanicolaou test (or “Pap test”) and treating the ailments that developed [6, 7].

Since 2006, two vaccines have been approved for use in Canada against HPV. One (Cervarix) protects against types 16 and 18 while the other (Gardasil) also protects against HPV types 6 and 11. These vaccines offer protection for at least eight years [8]. In 2007, the Canadian federal government provided the provinces and territories with $300 million to spend over 3 years for HPV immunization programs [9]. By 2009, every province and territory in Canada had an HPV vaccination program in place [10]. The creation and implementation of the programs are the responsibility of the individual provinces and territories. This results in eight distinct strategies throughout Canada, which can be seen in Table 1.

These strategies vary in the number of doses of the quadrivalent vaccine given (only two doses in British Columbia and Quebec, with three doses administered elsewhere), the grade of the girls who are given the vaccine (ranging from 4 to 10), and the coverage rate (49–86% within the first two years of the program) [10]. Although the implementation of these programs differs between provinces, they all share a common goal of reducing the impact of HPV on the Canadian population [11]. This is measured through the morbidity and mortality rates of HPV [11].

Since the vaccine's introduction, there have been a number of mathematical models assessing its impact on several populations. In terms of the cost-effectiveness of the vaccine, Brown and White used an optimal control model for vaccinating adolescent females and males in the United Kingdom [12]. Brisson et al. used a cohort model to estimate how many girls need to be vaccinated to prevent cervical cancer and genital warts in Canada [13]. In terms of the disease impact on a population, Barnabas et al. developed a transmission model to measure the impact of vaccinating against HPV-16 in Finland for both women and men [14]. Although it does consider the effects caused by HPV later in life, this model neglects to consider herd immunity. There are current clinical trials in British Columbia looking at the effect of taking two or three doses of the quadrivalent vaccine [15]. This will provide valuable information about the levels of protection provided by two or three doses. Llamazares and Smith? developed an epidemiological model that includes a widespread childhood vaccination program with voluntary adult vaccination [4]. The model is used as a preliminary investigation to determine whether provincial healthcare programs in Canada should fund adult HPV vaccination. The authors determined a threshold of eradication for the targeted types of HPV and critical efficacy and immunogenicity levels such that eradication is not possible at any coverage rate.

Here we develop a mathematical model to explore the current vaccination strategies across Canada as well as potential alternative strategies. These strategies are defined by the number of doses given, the grade of the girls the vaccine is given to, and the coverage rate achieved. Our model will provide similar insight into the effectiveness of two and three doses of the quadrivalent vaccine. This model also provides insight into what the programs should look like in order to achieve a desired outcome. This model provides a unique perspective in its evaluation of the provincial programs based on the grade of vaccination, number of doses given, and the necessary coverage rate. We address the following research questions: (1) Does the grade at which the girls are vaccinated significantly affect the outcome of the program? (2) What coverage rate must the provinces reach in order to reduce the impact of HPV on the Canadian population? (3) What are the implications of vaccinating with two versus three doses?

This paper is organized as follows.  Section 2 gives the model description and equations.  Section 3 details the analysis of the model.  Section 4 gives the expressions for critical thresholds of efficacy and probability of protection.  Section 5 explores how the parameters were estimated, the results on the infection when varying the grade, dosage and coverage rate, and the sensitivity analysis of the model.  Section 6 discusses the implications.

## 2. The Model

Our model is composed of 19 equations, 13 that describe the childhood vaccination strategy and six that describe the disease propagation through adults. The female children are broken up into seven grades (4 through 10). Within each appropriate grade class, there are unvaccinated (*C*_*iU*_, where 4 ≤ *i* ≤ 10) and vaccinated female children (*C*_*iV*_, where 4 ≤ *i* ≤ 10). In the adult portion of the model, women are broken up into unvaccinated uninfected susceptible women (*A*_*U*_), vaccinated uninfected susceptible women (*A*_*V*_), unvaccinated and infected women (*I*_*U*_), or vaccinated infected women (*I*_*V*_). Men are considered either uninfected susceptible (*M*) or infected (*N*).

Define

(1) f=cϵWpW1−ϵWpW+γ,♀=C4+C5U+C5V+C6U+C6V+C7U+C7V +C8U+C8V+C9U+C9V+C10U+C10V +AU+AV+IU+IV,♂=M+N.

Girls in grade 4 (approximately 9 years old) are described as

(2) $$\begin{array}{c} \frac{dC_{4}}{dt}=\pi_{W}-\left(1+\mu_{C}\right)C_{4}. \end{array}$$

For girls in grade *i*, where 5 ≤ *i* ≤ 10, we have

(3) dC(i+1)Udt=1−ϵpiCiU−1+μCCi+1U,dC(i+1)Vdt=ϵpiCiU+CiV−1+μCC(i+1)V.

Uninfected adult women are described as

(4) dAUdt=(1−ϕU)C10U+ξUIU−f(ϵWpW)AU −βWAUN♂−μAAU+ωAV,dAVdt=(1−ϕV)C10V+ξVIV+f(ϵWpW)AU −(1−ψ)βWAVN♂−μAAV−ωAV.

Infected adult women are described as

(5) dIUdt=ϕUC10U+βWAUN♂−ξUIU−μAIU+ωIV,dIVdt=ϕVC10V+(1−ψ)βWAVN♂−ξVIV−μAIV−ωIV.

Uninfected men are described as

(6) $$\begin{array}{c} \frac{dM}{dt}=\pi_{M}-\frac{\beta_{M}I_{U}M}{♀}-\frac{\beta_{M}I_{V}M}{♀}+\xi_{M}N-\mu_{A}M. \end{array}$$

Infected men are described as

(7) $$\begin{array}{c} \frac{dN}{dt}=\frac{\beta_{M}I_{U}M}{♀}+\frac{\beta_{M}I_{V}M}{♀}-\xi_{M}N-\mu_{A}N. \end{array}$$

A girl enters the model unvaccinated in grade 4 at a constant rate *π*_*W*_. At some grade between 4 and 10, a proportion of the girls become vaccinated at rate *ϵp*_*i*_ (where *p*_*i*_ is the proportion of girls given the vaccine in grade *i* and *ϵ* represents the vaccine efficacy for girls). When the girls enter grade 11, a proportion of unvaccinated girls (1 − *ϕ*_*U*_) grow up to become uninfected unvaccinated women (*A*_*U*_), while the remaining unvaccinated girls (*ϕ*_*U*_) are categorized as infected unvaccinated women (*I*_*U*_) to account for early childhood sexual activity [29]. A proportion of vaccinated girls (1 − *ϕ*_*V*_) grow up to become uninfected vaccinated women (*A*_*V*_), while the rest of the vaccinated girls (*ϕ*_*V*_) are categorized as infected vaccinated women (*I*_*V*_) to account for a proportion of girls who have contracted at least one of the targeted types before receiving the vaccine. Once in the adult stage (grade 11 to around the age of 26), all of the disease propagation takes place. Once the girl reaches grade 11, she is assumed to be sexually active.

**Figure 1:**
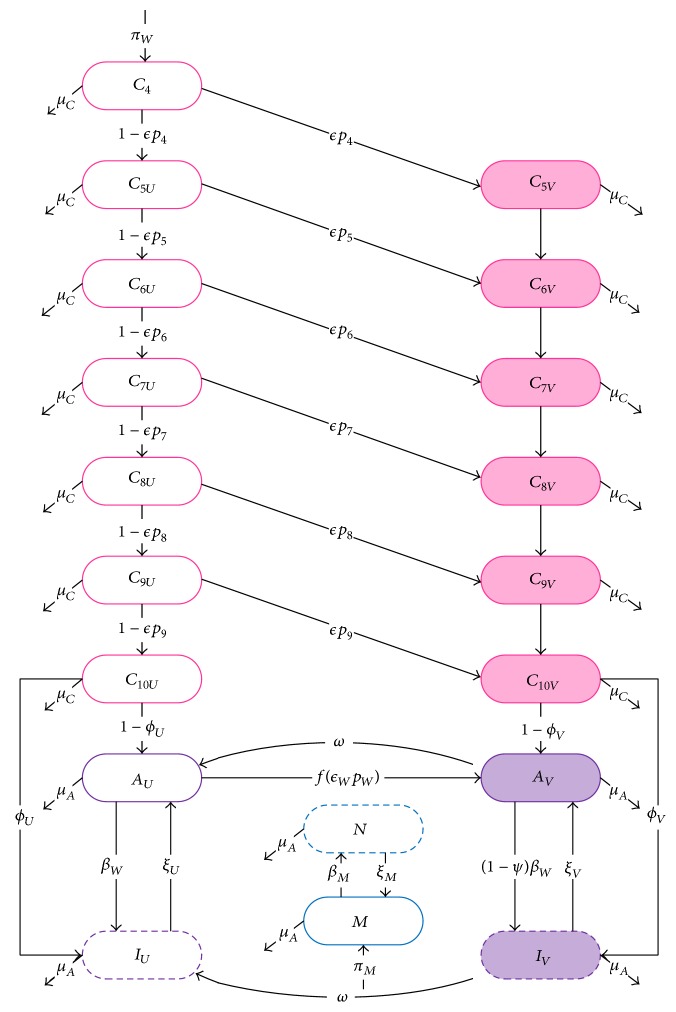
The model. Movement of individuals through childhood vaccination programs and the infection cycle as adults. Girls from grades 4 to 10 (pink, *C*_*i*_) can be vaccinated (shaded) depending on the proportion vaccinated (*p*_*i*_) and the efficacy of the vaccine (*ϵ*). Once the girls turn 16 (approximately grade 11), they enter adulthood (purple) where they may become infected (dashed) with HPV. Unvaccinated women (*A*_*U*_) can become vaccinated (*f*) or become infected (*I*_*U*_) with transmission rate *β*_*W*_ when they meet an infected man (*N*). Vaccinated adult women (*A*_*V*_) can also become infected (*I*_*V*_) but with a reduced transmission rate, due to the failure of the vaccine (*ψ*). Unvaccinated men (*M*) become infected upon contact with infected women, with transmission rate *β*_*M*_. The vaccine wanes at rate *ω*, while protection is lost at rates *ξ*_*U*_, *ξ*_*V*_. The mortality rate for children is *μ*_*C*_ and the leaving rate of adults is *μ*_*A*_. The parameters *ϕ*_*U*_, *ϕ*_*V*_ measure the degree of preexisting infection in children.

Unvaccinated adult women (*A*_*U*_) can become vaccinated at rate *f*(*ϵ*_*W*_ *p*_*W*_), where *ϵ*_*W*_ is the efficacy of the vaccine for adult women and *p*_*W*_ is the proportion of women who get the vaccine. The vaccine wanes at rate *ω*. Unvaccinated adult women become infected (*I*_*U*_) at rate *β*_*W*_ when coming into contact with an infected man (*N*). Vaccinated adult women (*A*_*V*_) can also become infected (*I*_*V*_) at rate (1 − *ψ*)*β*_*W*_, where *ψ* represents the ability of the vaccine to protect against all targeted types. Men enter the model as susceptible (*M*) through a constant rate *π*_*M*_ and stay in the model for approximately 10 years. Unvaccinated susceptible men (*M*) can become infected after sexual activity with an infected woman (*I*_*U*_ or *I*_*V*_) with transmission rate *β*_*M*_. Infection clears at rate *ξ*_*U*_ for unvaccinated women, at rate *ξ*_*V*_ for vaccinated women, and *ξ*_*M*_ for men. Each compartment also includes a natural leaving rate, *μ*_*C*_ or *μ*_*A*_, depending on child or adult status. The flow diagram is illustrated in Figure 1. Parameter descriptions, ranges, and sample values can be found in Table 2.

For this model, it is assumed that HPV is only heterosexually transmitted. Sexual partnerships are not explicitly modelled [30]. Female children are in grades 4 through 10, which are the years childhood vaccination can take place. It is assumed that vaccination only occurs in one year, that male vaccination is negligible, and that the proportion of female children vaccinated during other years is negligible. The rate at which the HPV infection is cleared is independent of previous infection status. The transmission from women to men is higher than the transmission from men to women [2]. Both women and men are active in the adult model for approximately 10 years because the vaccine is not recommended for women over the age of 26 [7]. We keep men in the model for the same length of time as women, on the grounds that, while there may be an age difference between young women and older men, such men are “aging out” with their sexual cohorts [4]. That is, they may choose new partners within this cohort (e.g., friends of existing partners), but we assume they do not revert to an even younger cohort after such a cohort ages out. (However, we have explored this assumption in more detail elsewhere [31] and shown that relaxing it has a negligible effect on the epidemic, so it is only included here for analytical convenience.)

The assumptions made about the vaccine are that the vaccine does not wane in children, that it may not protect 100% (this is based on the probability of protection and the efficacy), and that the vaccine does not protect someone who is already infected with the virus [32, 33]. The difference between a two- and three-dose schedule is the efficacy of the vaccine. Lastly, we do not consider disease-induced death since it does not play a role in removing sexually active individuals from the pool that we are considering (complications due to HPV, such as cervical cancer, are generally seen much later in life) [7, 34, 35].

## 3. Analysis

### 3.1. Stability of the Disease-Free Equilibrium

The disease-free equilibrium is

(8) C4¯,C5U¯,C5V¯,C6U¯,C6V¯,C7U¯,C7V¯,C8U¯,C8V¯,C9U¯,C9V¯, C10U¯,C10V¯,AU¯,AV¯,IU¯,IV¯,M¯,N¯,

where

(9) $$\begin{array}{c} \bar{C_{4}}=\frac{\pi_{W}}{1+\mu_{C}},\;\text{and}\;\text{we}\;\text{define}\;\bar{C_{4U}}=\bar{C_{4}},\;\;\bar{C_{4V}}=0. \end{array}$$

Then, for 5 ≤ *i* ≤ 10, we have

(10) $$\begin{array}{c} \bar{C_{iU}}=\frac{\left(1-\epsilon p_{\left(i-1\right)}\right)\bar{C_{(i-1)U}}}{1+\mu_{C}},\\ \bar{C_{iV}}=\frac{\epsilon p_{\left(i-1\right)}\bar{C_{(i-1)U}}+\bar{C_{(i-1)V}}}{1+\mu_{C}},\\ \bar{A_{U}}=\frac{\left(1-\phi_{U}\right)\bar{C_{10U}}}{f\left(\bar{\epsilon_{W}}\;\bar{p_{W}}\right)+\mu_{A}},\\ \bar{A_{V}}=\frac{f\left(\bar{\epsilon_{W}}\;\bar{p_{W}}\right)\bar{A_{U}}+\left(1-\phi_{V}\right)\bar{C_{10V}}}{\mu_{A}},\\ \bar{I_{U}}=0,\;\;\bar{I_{V}}=0,\\ \bar{M}=\frac{\pi_{M}}{\mu_{A}},\;\;\bar{N}=0. \end{array}$$

The Jacobian matrix for this model evaluated at the disease-free equilibrium is *J*_DFE_ = [*J*_DFE_^(1)^ | *J*_DFE_^(2)^ | *J*_DFE_^(3)^], where
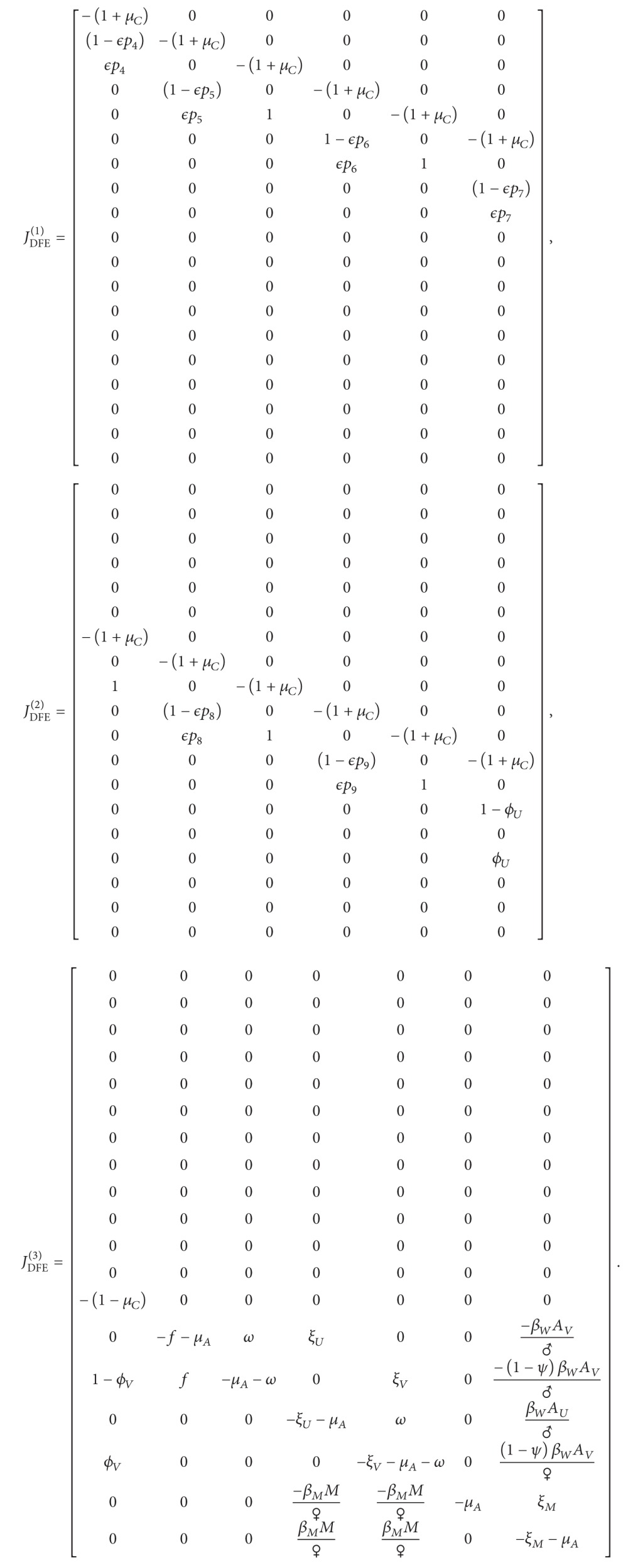
(11)The characteristic polynomial of the Jacobian is

(12) $$\begin{array}{c} \det\left(J-\lambda I\right)={\left(-1-\mu_{C}-\lambda \right)}^{13}\left(-\mu_{A}-\lambda \right)\det\left(H\right)\det\left(L\right), \end{array}$$

where

(13) H=−f−μA−λωf−μA−ω−λ,L=−ξU−μA−λωβWAU¯♂0−ξV−μA−ω−λ(1−ψ)βWAV¯♂βMM¯♀βMM¯♀−ξM−μA−λ.

Solving det⁡(*H*) = 0, we get

(14) $$\begin{array}{c} \lambda^{2}+\left(f+2\mu_{A}+\omega \right)\lambda +\mu_{A}\left(f+\mu_{A}+\omega \right)=0, \end{array}$$

which has only eigenvalues with negative real part.

Solving det⁡(*L*) = 0, we get

(15) $$\begin{array}{c} \lambda^{3}+\alpha \lambda^{2}+\chi \lambda +\rho =0, \end{array}$$

where (noting that $\bar{M}=♂$)

(16) α=3μA+ξU+ξV+ξM+ω,χ=3μA2+ξUξV+ω+ξUξM+ξV+ωξM +2μAξU+ξV+ξM+ω−1−ψβWβM  AV¯♀ −βWβMAU¯♀,ρ=μA3+μA2ξU+ξV+ξM+ω +μAξUξV+ω+ξUξM+ξV+ωξM +ξU(ξV+ω)ξM −1−ψβWβM  AV¯♀μA+ξU+ω −βWβMAU¯♀μA+ξV+ω.

In order to determine the stability of the disease-free equilibrium in the linearized system, we use the Routh–Hurwitz stability criterion. For our cubic characteristic polynomial (15), we have four Routh–Hurwitz conditions that must all be satisfied in order for the disease-free equilibrium to be locally stable:

(1) *α* > 0;
(2) *ρ* > 0;
(3) *χ* > 0;
(4) *α* · *χ* > *ρ*.

The first condition is satisfied, since all of the parameters are positive.

The second condition is our threshold. If *ρ* < 0, then we are guaranteed to have at least one positive real root of the characteristic polynomial. If *ρ* = 0, then we have a nonhyperbolic fixed point and must use stability theory to determine the stability of the disease-free equilibrium. We determine stability for the case when *ρ* = 0^+^ = *δ* > 0 (where *δ* is a small positive perturbation). We will show that *χ* > 0 and *αχ* > 0 in order to satisfy the Routh–Hurwitz criterion.

Define

(17) ⋄=ξU+ξV+ξM+ω♡=ξU(ξV+ω)+ξUξM+(ξV+ω)ξM.

Rearranging the original expression of *ρ*, we find

(18) (1−ψ)βWβMAV¯♀  =1μA+ξU+ωμA3+μA2⋄+μA♡   =1μA+ξU+ω−βWβMAU¯♀μA+ξV+ω   =1μA+ξU+ω+ ξUξV+ωξM−δμA3.

Substituting this into *χ*, we have

(19) χ=3μA2+2μA⋄+ ♡−1μA+ξU+ω ·μA3+μA2⋄+ μA♡−βWβMAU¯♀μA+ξV+ω  + ξU(ξV+ω)ξM−δβWβMAU¯♀−βWβMAU¯♀.

To require *χ* > 0, we need

(20) (μA+ξU+ω)3μA2+2μA⋄+ ♡   +βWβMAU¯♀μA+ξV+ω+δ  >μA3+μA2⋄+ μA♡+βWβMAU¯♀μA+ξU+ω   +ξUξV+ωξM,3μA3+2μA2⋄+ μA♡+3μA2ξU+2μAξU⋄+ ξU♡+3μA2ω   +2μAω⋄+ ω♡+(μA+ξV+ω)βWβMAU¯♀+δ  >μA3+μA2⋄+ μA♡+(μA+ξU+ω)βWβMAU¯♀   +ξUξV+ωξM,2μA3+μA2(⋄+3ξU+3ω)+2μAξU⋄+ ξU♡−ξU(ξV+ω)ξM   +2μAω⋄+ ω♡+(ξV−ξU)βWβMA¯U♀+δ>0.

Note that

(21) ξU♡−ξUξV+ωξM  =ξUξUξV+ω+ξUξM+ξV+ωξM   −ξUξVξM−ξUξMω>0.

It follows that *χ* > 0 if

(22) $$\begin{array}{c} \xi_{V}\geq \xi_{U}, \end{array}$$

that is, if the recovery rate from infection is faster for vaccinated individuals (or at least not worse), which we expect to be the case.

When *ρ* = 0^+^, the Routh–Hurwitz conditions are satisfied, all roots have a negative real part, and our system is stable at the disease-free equilibrium. Since *α* > 0 and *χ* > 0 when *ρ* = 0^+^, then *ρ* = 0 is our threshold of stability.

**Figure 2:**
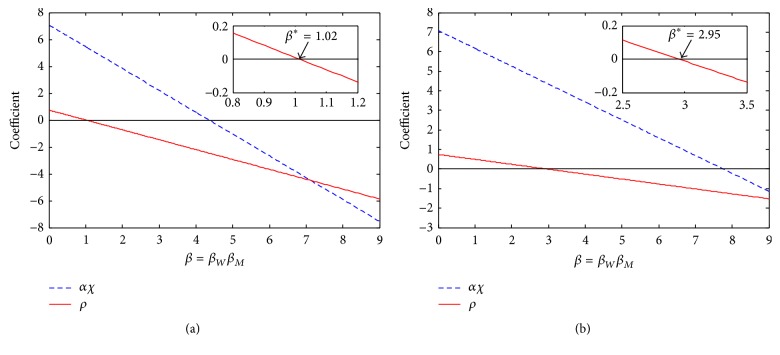
Stability when *ρ* > 0. Panel (a) shows the scenario with no vaccination, whereas panel (b) shows the scenario with 100% child and 100% adult vaccination. The dashed line represents *αχ*, and the solid line represents *ρ*. These are the two conditions that determine the stability of the disease-free equilibrium for our system. The disease-free equilibrium is stable in the region where the dashed line is above the solid line and when the solid line is above zero. This region is shown in more detail in the inset graph. We compare these conditions to *β* = *β*_*W*_ *β*_*M*_ since these parameters always show up in this fashion. The *β* ^∗^ values indicated in the inset graphs are the critical *β* values which determine when the disease-free equilibrium becomes unstable. Since panel (a) includes no vaccination, this *β* ^∗^ indicates that the actual value of *β* within the Canadian population must be at least 1.02 since HPV is an epidemic and has not been eradicated. Since panel (b) depicts the scenario with 100% childhood and 100% adult vaccination, if the actual *β* exceeds 2.95, then, even with 100% vaccination, we cannot eradicate HPV.

We used the product *β* = *β*_*W*_ *β*_*M*_ to numerically examine the Routh–Hurwitz coefficients *ρ* and *αχ*.  Figure 2(a) shows the scenario before vaccination while Figure 2(b) shows the scenario with 100% children and 100% adult vaccination. Note that the grade of childhood vaccination does not change the results. The region of stability does not significantly change depending on the efficacies (*ϵ*_*C*_ and *ϵ*_*W*_) or the probability of protection (Ψ). In Figure 2, *ρ* is represented by the solid red line while *αχ* is represented by the dashed blue line. When *ρ* > 0, *αχ* > 0. Since *α* > 0, then *χ* > 0. This satisfies the first three Routh–Hurwitz stability criteria.

The disease-free equilibrium is stable only if *ρ* > 0 and *αχ* > *ρ*. The value of *β* ^∗^ indicated on the inset graph of Figure 2 shows the threshold of transmission between a stable and unstable disease-free equilibrium. These values change depending on the coverage rate. However, if *β* < *β* ^∗^, then *ρ* > 0 and *αχ* > *ρ*. This shows that we can use *ρ* as a (local) threshold of stability. This threshold is discussed in greater detail in Section 3.2.

### 3.2. Basic Reproductive Number

The basic reproductive number *R*_0_ is a threshold that represents the average number of secondary infections caused by one infectious person in a completely susceptible population [36]. From the analysis in Section 3.1, the *R*_0_ threshold is

(23) R0=βWβM((1−ψ)(μA+ξU+ω)AV¯+(μA+ξV+ω)AU¯)♀[μA3+μA2⋄+ μA♡+ξU(ξV+ω)],

where $\bar{A_{U}}$ and $\bar{A_{V}}$ are the values at the disease-free equilibrium.

Knowing this threshold is significant, since it will tell us which parameters are involved in shifting the disease from persistence to eradication. This threshold will be used to determine which parameters have the greatest influence on *R*_0_, which in turn will give us insight into the most effective intervention strategies (Section 5.5).

## 4. Critical Thresholds

There are critical vaccine efficacies for both children (*ϵ* ^∗^) and women (*ϵ*_*W*_^∗^) and there is a critical probability of protection (*ψ*) that serves as a threshold where, even with 100% vaccination, the targeted types of HPV cannot be eradicated in the population. These values are determined by first setting *R*_0_ = 1 and rearranging for the desired parameter. In order to simplify the expression for *R*_0_, we rewrite the equilibrium values for our population in a general form. Note here that *k* ^∗^ represents the grade of childhood vaccination.

For 4 ≤ *k* ≤ 10, we find

(24) CkU¯=πW1+μCk−3 for  k≤k∗,CkU¯=πW1−ϵpk−11+μCk−3 for  k>k∗,CkV¯=0 for  k≤k∗,CkV¯=πWϵ1+μCk−3 for  k>k∗,AU¯=πWfpWϵW+μA1−μC7,AV¯=πWffpWϵW+μA1−μC7.

To determine the expression for *ϵ* ^∗^, we look at only childhood vaccination (i.e., no adult vaccination), which we get by setting *p*_*W*_ = 0. Since childhood vaccination only occurs during one year, then

(25) $$\begin{array}{c} p_{k}=\left\{\begin{array}{cc} 1, & \text{when}\;k=k^{*},\\ 0, & \text{when}\;k\neq k^{*}. \end{array}\right. \end{array}$$

Rearranging the expression *R*_0_ = 1 and solving for *ϵ* ^∗^, we find

(26) ϵ∗=1+μC7μA2βWβM(μA+ξU+ω)(1−ϕU)πWμA  − μA31+μC7♀(μA2+μA⋄+♡) ·βMβMμAμA+ξV+ω1−ϕUπWβMβM   −μA(1−ϕU)πW−1.

Noticing that ♀′ ≠ 0 (i.e., ♀ is not constant), we construct the following inequality from the definition of ♀. We know that

(27) $$\begin{array}{c} \pi_{W}-\mu_{A}♀\leq {♀}^{\prime }\leq \pi_{M}-\mu_{C}♀,\\ {♀}_{W}=\frac{\pi_{W}}{\mu_{C}}\leq \bar{♀}\leq \frac{\pi_{W}}{\mu_{A}}={♀}_{G}, \end{array}$$

where ♀_*W*_ is the size of the female population when there are only women and ♀_*G*_ is the size of the female population when there are only girls. This allows us to bound *ϵ* ^∗^ between

(28) $$\begin{array}{c} \epsilon^{*}\left({♀}_{W}\right)\leq \epsilon^{*}\left(\bar{♀}\right)\leq \epsilon^{*}\left({♀}_{G}\right). \end{array}$$

If *ϵ* < *ϵ* ^∗^(♀_*W*_), then 100% coverage rate of girls will not be sufficient for eradication, since *R*_0_ > 1. If *ϵ* > *ϵ* ^∗^(♀_*G*_), then—with a sufficient coverage rate—it is possible for female-only vaccination to eradicate the targeted types of HPV. If *ϵ* is between the bounds, we cannot accurately predict the outcome of the vaccine intervention.

The critical adult efficacy occurs when there is no childhood vaccination but there is 100% adult vaccination. Using a similar approach to the critical childhood efficacy, we find

(29) ϵW∗=(1+γ)βWβMπW(μA+ξU+ω)(1−ϕU)μA− μAD=l·D(c−μA)−βWβMπW(μA+ξU+ω)=lllll·1−ϕU1−ψc−μA−1,

where *D* = (1 + *μ*_*C*_)^7^♀*μ*_*A*_(*μ*_*A*_^3^ + *μ*_*A*_^2^⋄+ *μ*_*A*_ *♡* + *ξ*_*U*_(*ξ*_*V*_ + *ω*)). *ϵ*_*W*_^∗^ is bounded as in

(30) $$\begin{array}{c} \epsilon_{W}^{*}\left({♀}_{W}\right)\leq \epsilon_{W}^{*}\left(\bar{♀}\right)\leq \epsilon_{W}^{*}\left({♀}_{G}\right). \end{array}$$

The interpretation of *ϵ*_*W*_^∗^ is similar to that of *ϵ* ^∗^.

## 5. Numerical Simulations

### 5.1. Transmission

Figure 2(a) shows the region of stability when no vaccination occurs. If the transmission in Canada is below *β* ^∗^ = 1.02, then the disease would be eradicated without any intervention. Since HPV is endemic throughout the world and has not become eradicated, we conclude that the actual transmission parameters must be greater than 1.02 (assuming all other parameters used in the simulation are correct).

Figure 2(b) shows the region of stability for 100% childhood and 100% adult vaccination. As long as the transmission is lower than *β* ^∗^ = 2.95, then the introduction of the vaccine has the ability to eradicate HPV. However, if the transmission is above 2.95, then even 100% vaccination cannot eradicate HPV. These values allow us to estimate a range of likely transmission values, which are currently unknown.

### 5.2. Estimation of Parameters

The probability of protection, *ϕ*, was estimated by including the transmission of the targeted HPV types and an estimate for the proportion of girls who become sexually active before grade 11 (30%) [25]. Assuming all of the sexually active girls came into contact with someone able to transmit one of the targeted HPV types, the infection for this grade class would spread at a rate of 30% × *β*, giving a range of 0 ≤ *ϕ* ≤ 0.3.

The recovery rate was found by determining the average infectious period for both high- and low-risk types of HPV (1/*ξ*) and solving for *ξ* [26, 27].

### 5.3. Varying Grade

**Figure 3:**
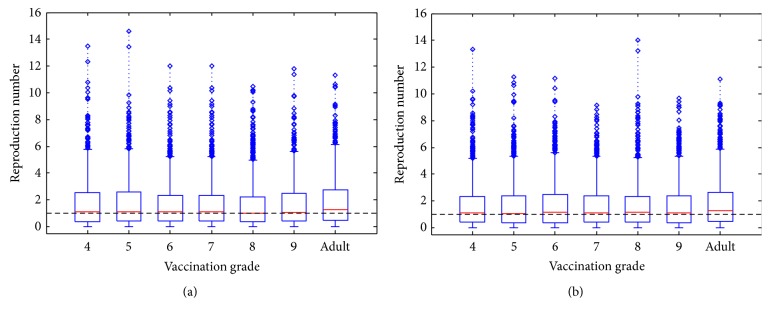
Effect of grade and dose. *R*_0_ values based on the grade of vaccination and the number of doses. Panel (a) represents the effects of two doses and panel (b) represents three doses. The grade of vaccination is controlled by forcing *p*_*i*_ = 0 except for the given grade. The number of doses is defined by different ranges of *ϵ* and *ϵ*_*W*_. The horizontal line represents the threshold for eradication, *R*_0_ = 1. For a childhood-only program, the median *R*_0_ is close to 1 (i.e., HPV types 6, 11, 16, and 18 are candidates for eradication), independent of the number of doses given. This contrasts with an adult-only vaccination program, where the median *R*_0_ is always above 1 (i.e., the disease will persist). All other parameters used the ranges in Table 2.

We looked at the significance of vaccinating girls at different ages by constructing a box plot as seen in Figure 3. For a specific grade, the coverage rate was varied between 0 and 100%. In Figures 3(a) and 3(b), the vaccine efficacy in adults was used to represent two (50–96%) and three (71–88%) doses, respectively. Latin Hypercube Sampling was used to sample parameter values from the given ranges in Table 2 using a uniform distribution. Latin Hypercube Sampling is a statistical sampling method in which parameters are assigned a range of values, and a distribution of plausible collections of these parameter values are created [37]. *R*_0_ was calculated 1000 times (diamonds) per grade. Note that only one grade (or adult) is considered during each run; for example, a box plot representing grade 4 only takes into account vaccinating girls in grade 4 and does not include vaccinating any other grade or adults. The thick red horizontal lines represent the median value of *R*_0_, while the box indicates the location of the upper and lower quartiles.

Comparing the median values in Figures 3(a) and 3(b), we notice that when girls are between grades 4 and 10, the values are all around 1, whereas the median *R*_0_ is always above one for adult vaccination regardless of the number of doses given. The upper quartile values and lower quartile values also follow this trend, where the childhood vaccination values all have a similar range that is noticeably smaller than when adult vaccination occurs.

**Figure 4:**
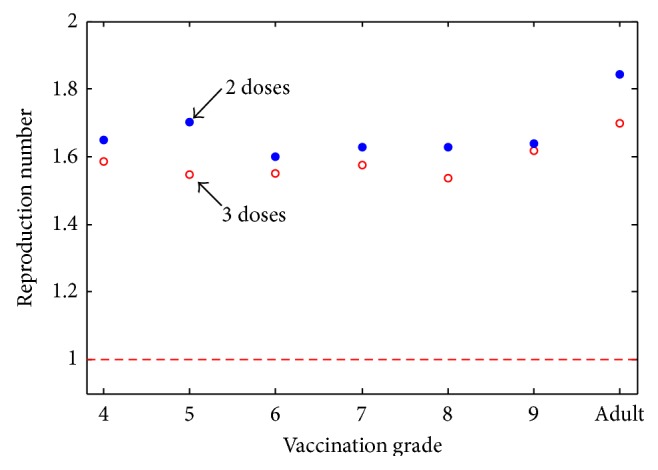
Effect of mean *R*_0_ values based on the grade of vaccination. For each grade of vaccination, the mean *R*_0_ was calculated from a run of 1000 parameters chosen using Latin Hypercube Sampling. The number of doses is defined by different ranges of *ϵ* and *ϵ*_*W*_. The dashed red line indicates the threshold *R*_0_ = 1. The dosing program (either two or three doses) that leads to the lowest mean *R*_0_ is not consistent nor does it show a grade-related trend. For a childhood-only program, the mean *R*_0_ is always above 1, independent of the number of doses given. For an adult-only vaccination program, the mean *R*_0_ is significantly higher, suggesting that a childhood-only vaccination program will be more successful than an adult-only one, even if neither leads to eradication.

**Figure 5:**
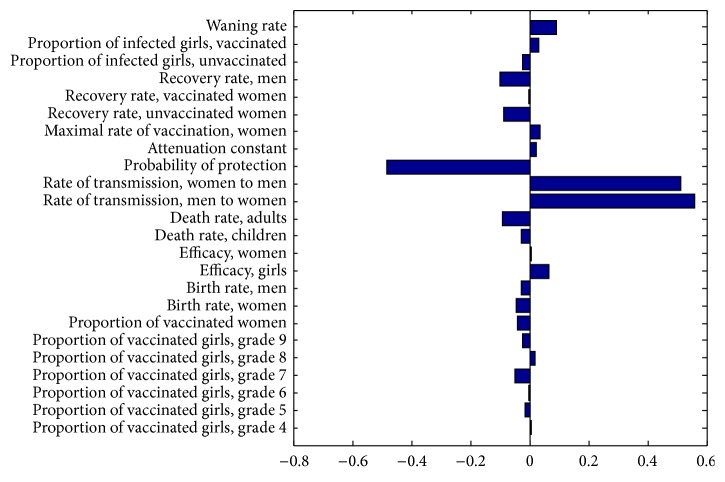
Partial rank correlation coefficient sensitivity analysis on *R*_0_ for all parameters. Here *R*_0_ is most sensitive to the transmission of HPV from men to women, *β*_*W*_, and from women to men, *β*_*M*_. *R*_0_ is also sensitive to the degree of protection, *ψ*. All ranges are as in Table 2.

Figure 4 shows the mean *R*_0_ values, rather than the median. While there is more variation between grades, vaccinating with 3 doses is always superior to vaccinating with 2 doses. Due to transmission rates (see Figure 5), neither form of vaccination will lead to eradication. However, childhood vaccination is always superior to adult vaccination.

### 5.4. Varying Dosage

Comparing Figures 3(a) and 3(b), we notice that the *R*_0_ values are generally smaller in the case of three doses, which is expected since the vaccine is less effective on a two-dose schedule. However the difference is minimal. This can also be validated by Figure 5, which shows a low correlation between *ϵ* or *ϵ*_*W*_ and *R*_0_.

Figure 4 shows the difference in the mean *R*_0_ when using a two-dose versus a three-dose regime. Once again, having three doses is clearly superior to two, but the difference is not significant.

### 5.5. Sensitivity to Variations

**Figure 6:**
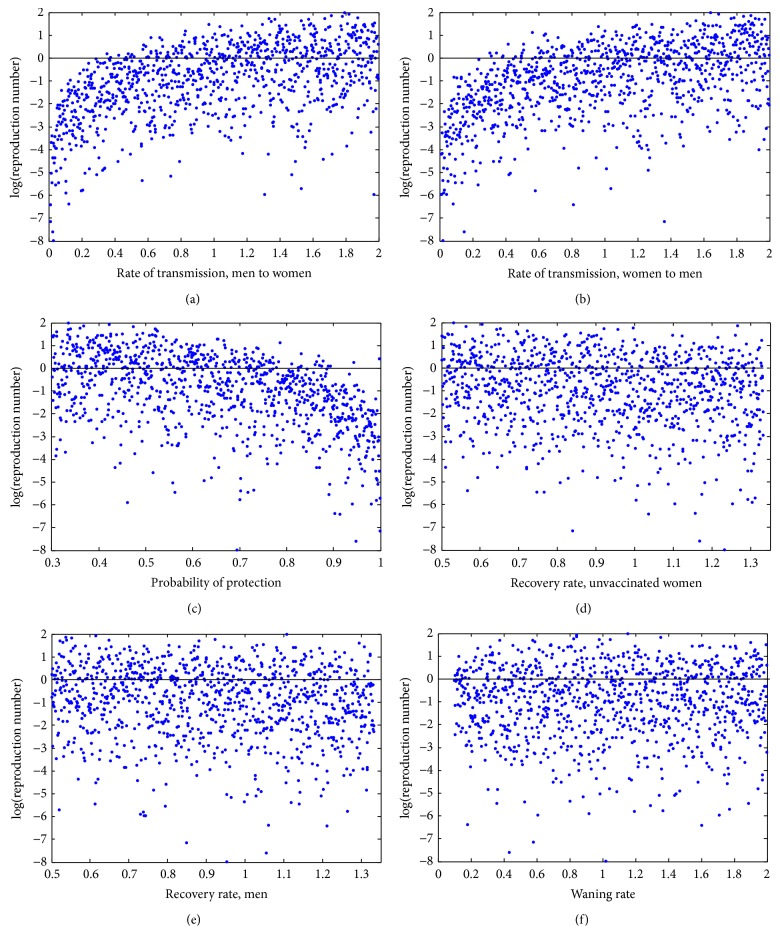
Latin Hypercube Sampling output for parameters of interest. Each graph shows the sampled parameter values from the given range on the horizontal axis and its degree of correlation between the sampled value and the value of *R*_0_. The increasing trend seen in the top two panels indicates a high degree of correlation between the values of *β*_*W*_ or *β*_*M*_ and their influence on *R*_0_. The next most influential parameter is the probability of protection (*ψ*), which has the opposite trend. Other parameters of interest (*ξ*_*U*_, *ξ*_*V*_, *ω*) are included for completeness but have only a very weak correlation with *R*_0_.

Since the true value of each parameter may fluctuate, we explore the sensitivity of *R*_0_ to the parameter values in Table 2 using Latin Hypercube Sampling. This in turn allows us to use partial rank correlation coefficients to rank the parameters in terms of their influence on *R*_0_, be it a positive or negative influence. Figure 5 shows the partial rank correlation coefficient sensitivity analysis on *R*_0_. The parameters were varied for 1000 runs. Here *R*_0_ is most sensitive to the transmission of HPV from men to women, *β*_*W*_, and from women to men, *β*_*M*_. The next influential parameter is the probability of protection, *ψ*. Notice that the coverage rates (*p*_*i*_, where 4 ≤ *i* ≤ 10, and *p*_*W*_) and vaccine efficacy (*ϵ* and *ϵ*_*W*_) do not significantly affect the value of *R*_0_.  Figure 6 shows the output of the Latin Hypercube Sampling for the three most influential parameters as well as some other parameters of interest: the recovery rates and the waning rate. Note the extremely weak correlation between the latter parameters and *R*_0_.

### 5.6. Varying Coverage Rate

**Figure 7:**
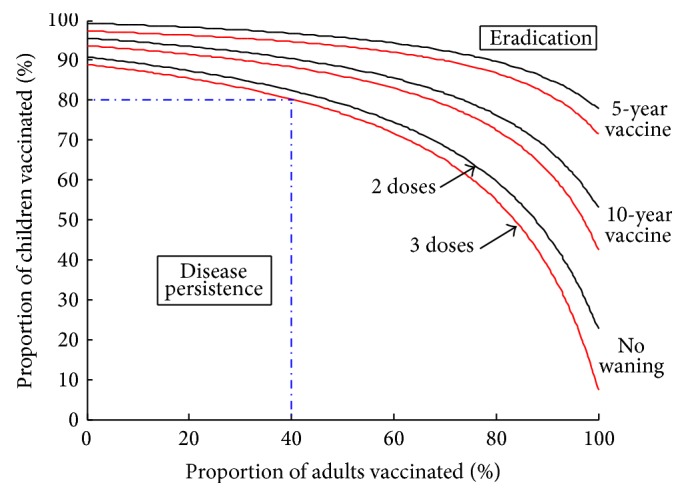
Thresholds of eradication for targeted types of HPV dependent on coverage rates of children and adults. The three-dose curve uses a childhood efficacy of 99% and an adult efficacy of 84.1%. The two-dose curve uses a childhood efficacy of 97% and an adult efficacy of 80.9%. Other parameters are given in Table 2. If no adult vaccination is undertaken, then a childhood vaccination program must cover 90% with three doses and 88% with two doses if the vaccine does not wane. If 80% of children are vaccinated with three doses of a nonwaning vaccine, then the eradication of targeted types can only be achieved if at least 40% of adults are also vaccinated; these requirements become harsher as the number of doses decreases or as the waning of the vaccine increases.

The coverage rate required to eliminate the targeted high-risk HPV types can be seen in Figure 7. Above the curves is the region where *R*_0_ < 1 and the targeted types are theoretically eradicated, whereas below the curves is the region where the disease persists in the population. The curves indicate when *R*_0_ = 1 for either two doses (lower red curves) or three doses (upper black curves) for different values of the waning rate. As expected, the curve for two doses is higher than three, since the efficacy for two doses is slightly lower. The targeted types can be eradicated if childhood vaccination is supplemented with significant adult vaccination. However, as the waning rate of the vaccine increases, the window for eradication shrinks, requiring a significant amount of both childhood and adult vaccination.

A childhood-only vaccination program can theoretically eradicate the targeted HPV types with either two or three doses, as long as the appropriate coverage rate is achieved (Figure 7). For example, if 80% of children are vaccinated with a 3-dose vaccine that does not wane (the lowest of the curves), then at least 40% of adults must be vaccinated to achieve eradication of targeted types. These requirements become harsher as the number of doses decreases or as the vaccine wanes. Note that these curves do not change with the grade of childhood vaccination.

## 6. Discussion

Based on the results of the model, we conclude that, with sufficient childhood and adult vaccination, it is theoretically possible to eradicate targeted HPV types. We also determine that the grade of vaccination before sexual debut does not significantly affect the prevalence of the targeted HPV types for the Canadian population. Comparing Figures 3(a) and 3(b), we observe that the median value of *R*_0_ is always close to one when children are vaccinated, while the median value of *R*_0_ is above one when only adult women are vaccinated, independent of the number of doses given. This suggests that the targeted types are candidates for eradication if significant childhood vaccination can be achieved, whereas eradication is less likely from adult-only vaccination.

Looking only at childhood-only vaccination, there does not seem to be a large difference between the grades. From a mathematical standpoint, it is easy to understand why the grade of vaccination does not matter in the model, since there is no impact in terms of the disease dynamics, whether girls are vaccinated in grade 4 or 10. The large jump is due to the change in vaccine effectiveness for those previously exposed to the considered HPV types, as well as the possibility of overvaccinating women, since they remain within the vaccination cohort for 10 years, whereas children are only vaccinated within a single year. This is important to determine because it shows there are likely no underlying grade-dependent trends. However, from an epidemiological standpoint, we know that the vaccine has little to no impact on those individuals who have a previous infection with a targeted type [32, 33]. Therefore public vaccination strategies should choose the grade of vaccination based on epidemiological and vaccination program limitations.

Assuming that the number of doses only changes the efficacy of the vaccine, the evidence from this model suggests that the number of doses does not significantly change the outcome of the vaccine strategy. Figure 7 shows that the possibility of eradication of targeted HPV types exists with either two or three doses, independent of the grade the childhood vaccine is given in. This evidence follows the findings by several clinical studies that suggest that two doses of the bivalent vaccine may protect just as well as three doses [5, 15, 22]. The higher the coverage rate, the higher the success of the vaccine program (Figure 7). Based on this evidence, we suggest each province chooses the number of doses based on cost or ease of program implementation, as long as an appropriate coverage rate is matched to achieve the desired level of infection in the community.

The value of *R*_0_ is mainly affected by the ability of the targeted HPV types to transmit and the ability of the vaccine to prevent the infection. Thus the most effective way to decrease *R*_0_ is to decrease the transmission of the targeted types of HPV. This could be done through increasing condom use, reducing the number of sexual partners, or increasing the heterogeneity of the sexual contact network of the population [16, 38]. Since HPV has been infecting humans for millions of years and has not been eradicated, the real transmission rate in the Canadian population must be greater than 1.02. By studying the critical transmission value for 100% childhood and adult vaccination, we know that if the actual transmission rate is greater than 2.95, then, even with 100% vaccine coverage, the targeted HPV types could not be eradicated. Looking at the stability of the disease-free equilibrium based on the transmission is reasonable since the transmission parameters are the only two parameters that significantly affect the value of the basic reproductive number, as shown by the sensitivity analysis.

We chose the estimates for the efficacies (*ϵ* and *ϵ*_*W*_) based on clinical evidence of both the bivalent and the quadrivalent vaccine. Obviously, a single vaccine program will only use either the bivalent or the quadrivalent vaccine. Since this model does not differentiate between different HPV types and the efficacies for either vaccine do not differ greatly, the numerical results hold for either vaccine. Through sensitivity analysis, using larger ranges of the efficacy than observed for either vaccine, we see in Figure 5 that it is not important to narrow down the range of efficacies since neither *ϵ* nor *ϵ*_*W*_ affects the value of *R*_0_ significantly. In order to estimate the efficacy of a two-dose schedule of the quadrivalent vaccine, we used the efficacy of the bivalent vaccine from the Costa Rica clinical trial [22]. This model should be updated once better data is available for the efficacy and effectiveness of a two-dose quadrivalent HPV vaccine.

It is important to note that our model has several limitations. In terms of vaccination programs, the model does not include catch-up programs unless included in the initial population conditions. Our model does not allow the vaccine program to differ amongst individuals. Each person receiving the vaccine with either two or three doses must complete the regimen within one year. The possibility of male vaccination is not included, although it is now approved by Health Canada [1, 5, 11]. We limit transmission to that of heterosexual couples. The model does not differentiate in HPV 6, 11, 16, or 18 or include any other HPV types. This model does not include immigrating (infectious) adults. We have also not ruled out the possibility of a backward bifurcation, meaning the disease may persist even for *R*_0_ < 1, complicating eradication efforts.

From the base adult model, we assume that men who have sexual relations with women in the sexually active cohort (i.e., women who are eligible for adult vaccination) do not continue to find new partners in this age group as time goes on. The sexually active cohorts of men and women are linked only for the time (approximately 10 years) that adult women are sexually active and eligible for vaccination [4]. Note, however, that we have previously shown that this cohort assumption is negligible [31].

Since the provincial vaccination programs are already in place and each province has chosen their dosing schedule (although it is possible they may change), we recommend that all of the provinces focus on increasing their coverage rate for both the public vaccination of girls and private vaccination of women to at least 80%. This number is realistic for many provinces, since several have already reached this goal (Quebec, Nova Scotia, Newfoundland, and PEI). For the other provinces, increasing their coverage rate may be accomplished by creating public-education campaigns or may involve making protocols similar to vaccines necessary to attend school such as those for Hepatitis B [17, 39].

Our model suggests that the grade of vaccination does not affect the outcome of the vaccination program. Therefore we suggest provinces vaccinate girls as early as possible to avoid vaccine failure due to previous infection. We also recommend that the number of doses should be chosen for optimal uptake. The main focus should be on obtaining large enough coverage rates for children and/or adults in order to achieve the desired outcome: using the vaccine to reduce the prevalence of HPV types 6, 11, 16, and 18 in the population.

## Figures and Tables

**Table 1:** Overview of provincial vaccination programs.

Strategy	Province(s)	Grade	Doses	Coverage rate	Reference
1	NWT	4	3	Unknown	—

2	QU	4, 9	2, 1 (last)	81–86%	[16]

3	AB	5	3	50–60%	[16]

4	BC	6, 9	2	62%	[16, 17]

5	NL	6, 9	3	85%	[16]

6	MB	6	3	52–61%	[16]
6	NU	6	3	Unknown	—
6	PE	6	3	85%	[16]
6	SK	6	3	58–66%	[16]
6	YK	6	3	Unknown	—

7	NS	7	3	85%	[16, 18]
7	NB	7	3	Unknown	—

8	ON	8	3	49–59%	[16, 19, 20]

**Table 2:** List of parameters and state variables.

Parameter	Description	Range	Sample value	Reference
*C*_*iU*_	Unvaccinated children in grade *i*	*(State variable) *		
*A*_*U*_	Unvaccinated adult women	*(State variable) *		
*C*_*iV*_	Vaccinated children in grade *i*	*(State variable) *		
*A*_*V*_	Vaccinated adult women	*(State variable) *		
*I*_*U*_	Uninfected adult women	*(State variable) *		
*I*_*V*_	Infected adult women	*(State variable) *		
*M*	Uninfected men	*(State variable) *		
*N*	Infected men	*(State variable) *		
*π*_*W*_, *π*_*M*_	Birth rate for women, men	50–100 people	100	[4]
*μ*_*C*_	Mortality rate for children	1/50–1/90 year^−1^	1/90	1/avg. time in compartment
*μ*_*A*_	Leaving rate for adults	1/12–1/8 year^−1^	1/10	1/avg. time in compartment
*ϵ*	Vaccine efficacy in female children	93.3–99.8% 2 doses	0.97	[3]
		93.3–99.8% 3 doses	0.99	[3]
*ϵ*_*W*_	Vaccine efficacy in adult women	50.2–96.3% 2 doses	0.809	[3, 5, 21–24]
		71.1–87.7% 3 doses	0.841	[3, 5, 21–24]
*p*_*i*_, where 4 ≤ *i* ≤ 9	Proportion of children vaccinated within the corresponding grade	0–100%		[16, 17, 19, 20]
*p*_*W*_	Proportion of previously unvaccinated adult women vaccinated	0–100%		[16, 17, 19, 20]
*β*_*W*_	Transmission of infection of a woman by an infected man	0.01–2 year^−1^	0.77	[2]
*β*_*M*_	Transmission of infection of a man by an infected woman	0.01–2 year^−1^	0.77	[2]
*ϕ*_*U*,*V*_	Proportion of infected girls at age 15 unvaccinated, vaccinated	0–30%	10%	[25]
*ψ*	Probability of protection against all targeted types	0.3–1	0.44	[23]
*ξ*_*U*,*V*,*M*_	Recovery rate for unvaccinated women, vaccinated women, men	1/2–4/3 year^−1^	2/3	[26, 27]
*c*	Attenuation constant	0–0.3 year^−1^	0.15	[4]
*γ*	Maximal possible rate of adult vaccination	0–0.2	0.1	[4]
$f(\bar{\epsilon },\bar{p})=\frac{c\;\bar{\epsilon_{W}}\;\bar{p_{W}}}{1-\bar{\epsilon_{W}}\;\bar{p_{W}}+\gamma }$	Rate at which unvaccinated women are vaccinated			[4]
*ω*	Waning rate	0.2–1 year^−1^ 2 doses	0.5	[28]
		0.1–0.5 year^−1^ 3 doses	0.333	[28]
